# The role of epigenetic and epitranscriptomic modifications in plants exposed to non-essential metals

**DOI:** 10.3389/fpls.2023.1278185

**Published:** 2023-12-04

**Authors:** Jagna Chmielowska-Bąk, Iain Robert Searle, Theophilus Nang Wakai, Magdalena Arasimowicz-Jelonek

**Affiliations:** ^1^ Department of Plant Ecophysiology, Institute of Experimental Biology, Faculty of Biology, Adam Mickiewicz University, Poznań, Poland; ^2^ Discipline of Molecular and Biomedical Sciences, School of Biological Sciences, The University of Adelaide, Adelaide, SA, Australia; ^3^ Department of Biochemistry, Faculty of Science, University of Bamenda, Bambili, Cameroon; ^4^ Covenant Applied Informatics and Communication - Africa Centre of Excellence (CApIC-ACE), Covenant University, Ota, Nigeria

**Keywords:** 5-methylcytosine, histone modifications, N6-methyladenosine, M6A, 8-hydroxyguanosine, 8-nitroguanosine

## Abstract

Contamination of the soil with non-essential metals and metalloids is a serious problem in many regions of the world. These non-essential metals and metalloids are toxic to all organisms impacting crop yields and human health. Crop plants exposed to high concentrations of these metals leads to perturbed mineral homeostasis, decreased photosynthesis efficiency, inhibited cell division, oxidative stress, genotoxic effects and subsequently hampered growth. Plants can activate epigenetic and epitranscriptomic mechanisms to maintain cellular and organism homeostasis. Epigenetic modifications include changes in the patterns of cytosine and adenine DNA base modifications, changes in cellular non-coding RNAs, and remodeling histone variants and covalent histone tail modifications. Some of these epigenetic changes have been shown to be long-lasting and may therefore contribute to stress memory and modulated stress tolerance in the progeny. In the emerging field of epitranscriptomics, defined as chemical, covalent modifications of ribonucleotides in cellular transcripts, epitranscriptomic modifications are postulated as more rapid modulators of gene expression. Although significant progress has been made in understanding the plant’s epigenetic changes in response to biotic and abiotic stresses, a comprehensive review of the plant’s epigenetic responses to metals is lacking. While the role of epitranscriptomics during plant developmental processes and stress responses are emerging, epitranscriptomic modifications in response to metals has not been reviewed. This article describes the impact of non-essential metals and metalloids (Cd, Pb, Hg, Al and As) on global and site-specific DNA methylation, histone tail modifications and epitranscriptomic modifications in plants.

## Introduction

Plants being sessile organisms are exposed to multiple environmental stimuli and have evolved sophisticated physiological response mechanisms to adapt. Generally, plant responses to stresses can be divided into either short-term processes to acclimate and endure, or long-term changes affecting future stress responses. Plants can store and recall information from a preceding stimulus thereby providing an imprint for future stress events (Galviz et al., 2020). It is postulated, that plants can even maintain a memory of previous stress exposure throughout their ontogenesis and later promulgate the memory into the next generation through complex epigenetic mechanisms (Bilichak et al., 2015). Here we define epigenetics as a heritable change in the pattern of gene expression that occur without an alteration in the DNA sequence (Iwasaki and Paszkowski, 2014). Known epigenetic factors that modulate gene expression in response to diverse stimuli include DNA cytosine methylation, histone modifications and small RNAs (sRNAs) (Bilichak et al., 2015).

DNA cytosine methylation is the most well-documented epigenetic modification. Nearly a century ago in 1925, Johnson and Coghill isolated and crystalized nucleic acids from *Mycobacterium tuberculosis* and first identified 5-methylcytosine (5mC). However, it was not until the dawn of molecular biology in the 1960’s that thorough investigation of DNA methylation broadly occurred in lower and higher organisms (Mattei et al., 2022). Since then, a range of increasingly sophisticated techniques were developed and employed for assessment of global and site-specific methylation including Methylation-Sensitive Amplified Polymorphism (MSAP), Methylation-Sensitive Amplified Fragment Length Polymorphism (MetAFLP), ELISA and Mass Spectrometry based methods, methylation specific PCR, Whole Genome Bisulfite Sequencing (WGBS) and most recently, PacBio and Oxford Nanopore sequencing (reviewed in Kurdyukov and Bullock, 2016). In plants, the cytosine base can be methylated at the 5’ position in the CG, CHG and CHH sequence contexts, wherein H stands for either C, T or A. Methylation in the CG context is most frequently observed, followed by CHG and CHH. The level of 5mC is regulated by methylation and demethylation machinery. *De novo* methylation occurs through RNA-directed DNA methylation pathway (RdDM), while methylation of cytosines in daughter DNA strands during the replication process is mediated by methyltransferase 1 (MET1) chromomethylase 2 and 3 (CMT2 and CMT3, respectively) or domains rearranged methylase 2 (DRM2) depending on the context. Decreases in cytosine demethylation can occur either non-enzymatically (passively) or enzymatically through the action of bifunctional DNA glycosylases, which initiate the base excision repair (BER) process. In Arabidopsis four bifunctional DNA glycosylases have been identified – Repressor Of Silencing (ROS1), Demeter (DME), Demeter-like protein 2 (DML2) and Demeter-like protein 3 (DML3) (Czajka et al., 2021; Kumar and Mohapatra, 2021). Plants are characterized by high levels of DNA 5mC when compared to other organisms. For example, vertebrate genomes comprise around 5% 5mC, while in plants, 5mC reaches on average 10%, with some species (e.g. rice or onion) exhibiting 35-50% methylation (Czajka et al., 2021; Varma et al., 2022). Methylation of DNA determines the structure of chromatin thereby modulating chromatin accessibility to the transcriptional machinery and thus influences expression of specific genes. Formation of 5mC in gene promoter regions is in general, associated with decreased expression, while methylation of the coding region can have varying effects. It was suggested that cytosine methylation exhibits regulatory roles during developmental processes, stress responses and is involved in the inactivation of transposable elements (TE) (Czajka et al., 2021; Kumar and Mohapatra, 2021).

It is worth mentioning that DNA 5mC can be enzymatically converted to hydroxymethylcytosine (5hmC) and further modified into formylcytosine (5fC) and carboxycytosine (5cadC). However, 5fC and 5cadC have not been detected in significant quantities in studied plant genomes and therefore are likely not to exhibit a regulatory role (Varma et al., 2022). In the case of hmC, some reports are ambiguous and others conflicting. Results using enzymatic radiolabeling, mass-spectrometry and thin-layer chromatography indicated that 5hmC is not present in DNA isolated from Arabidopsis plants (Erdmann et al., 2015). In contrast, application of liquid chromatography-tandem quadrupole mass (QQQ) spectrometry with multiple reaction monitoring (LC-MRM) revealed low levels of hmC in the genomes of some plant species (Varma et al., 2022). DNA can be also methylated at N6 position in adenine (N6mA) and is predominant in fungi, ciliates and green algae. Intriguingly, it was also detected in several plant species including Arabidopsis and important crops like, rice, wheat and soybean (Jiménez-Ramírez et al., 2022; Varma et al., 2022). However, reports on N6mA’s regulatory roles in plants are still scarce.

Apart from 5mC, chromatin remodeling can be also driven by histone-associated changes e.g. core histone removal and replacement with specialize histone variants or addition of covalent chemical modifications to histone tails. Histone tail modifications include addition of methyl, acetyl and phosphate functional groups or proteins, like ubiquitin and small ubiquitin-like modifier (SUMO). Histones are usually modified on the N-terminal tail, although C-terminal tail modifications are also reported (Deal and Henikoff, 2011; Zhao et al., 2019). The effects of histone modifications can have varying effects on the state of chromatin and associated expression of genes, depending on their type, localization and combinations. For instance, mono- and demethylation of lysine at the 9^th^ position of the histone 3 (H3) tail, (H3K9me1 and H3K9me2) and trimethylation of lysine 27 (H3K27me3) are associated with repression of the transposable elements. In contrast, histone acetylation is related to chromatin relaxation and transcriptionally active states (Deal and Henikoff, 2011; Zhao et al., 2019)In addition to the epigenetic mechanisms, plants responses to stresses can be modified on the epitranscriptomic level. While epigenetics is related to chemical modifications of DNA, epitranscriptomics is associated with covalent chemical modifications of RNA (Saletore et al., 2013). The most widespread and studied modification of ribonucleotides is methylation of adenosine at N6 position (m6A). This modification is found in 70-100% mRNA transcripts depending on the plant species and organelle (Wan et al., 2015; Wang et al., 2017). In addition, it can occur at multiple sites on a transcript (Li et al., 2014). Studies on mutants deficient in RNA adenosine methylation or demethylation machinery revealed that m6A is crucial for plant development and stress responses (reviewed in Tang et al., 2023). It was also evidenced in Arabidopsis and rice that RNA 5-methylcytosine (m^5^C) is involved in developmental processes and responds to unfavorable environmental conditions, including oxidative stress and heat (David et al., 2017; Zhang et al., 2020). Modified ribonucleotides can also be formed because of reactive oxygen and/or nitrogen species action, ROS and RNS, respectively. The most frequent ROS-dependent RNA modification is 8-hydroxyguanosine (8-OHG), while RNS-dependent modification involves 8-nitorguanosine (8-NO_2_-G). Some studies also demonstrate changes in 8-OHG and 8-NO_2_-G levels in response to stresses (reviewed in Chmielowska-Bąk et al., 2019). However, their exact role is still to be elucidated.

In general, epigenetic modifications can contribute a long-lasting adjustment to the environmental changes, while epitranscriptomic modifications are perceived as a more rapid response. The data for plant epigenetic and epitranscriptomic responses under stress conditions is gradually expanding, with some comprehensive reviews on DNA methylation response under drought, osmotic stress, salinity, heat, cold and nutrient deficiency were recently published (Ashapkin et al., 2020; Oberkofler et al., 2021). However, information on the epigenetic and epitranscriptomic changes driven by metals is still scarce, which is an oversight given the environmental contamination with metals contributes to the reduction of agricultural productivity and decreased food safety. In particular, non-essential metals, such as cadmium (Cd), lead (Pb), aluminum (Al) and mercury (Hg), pose a serious threat due to their high toxicity even by relatively low concentrations. Common symptoms of metal toxicity in plants include reduction of root growth and leaf area, root swelling and chlorosis (Riyazuddin et al., 2021). Exposure to metals leads to decreased chlorophyll levels, alteration of chloroplast ultrastructure and inhibition of enzymes engaged in the photosynthesis leading to reduced photosynthesis efficiency. Furthermore, plants exposed to metals frequently show alerted mineral composition (Riyazuddin et al., 2021; Noor et al., 2022). Metal toxicity depends on a range of mechanisms at the cellular level. The most-reported and recognized mechanism is the production of ROS thereby leading to plant oxidative stress (Noor et al., 2022). Metals can bind to functional groups of proteins, changing the protein conformation and affecting protein folding processes which can result in protein aggregation. On a general view, proteins are considered to be the main targets of heavy metals either by complexation or by displacement of essential ions in metalloproteins. Harmful effects of heavy metals and metalloids can also occur by their ability to compete with residual ions that act as cofactors or ligands for enzymes (Chmielowska-Bąk et al., 2013; Tamás et al., 2014; Rahman and Singh, 2019). Metal stress can lead to the development of various symptoms of genotoxicity. This includes decreased mitotic index and higher frequency of chromosomal aberrations e.g. stickiness, bridges, fragmentation and formation of micronucleus (Dutta et al., 2018).

The aim of the present article is to comprehensively review the literature on epigenetic and epitranscriptomic changes in plants driven by metals, with special emphasis on their role in metal tolerance. The review focuses on the non-essential metals, which pose the most serious threat for the environment, plant and human health – cadmium (Cd), lead (Pb), mercury (Hg) and aluminum (Al). In addition, due to the significance for human health the impact of one metalloid, arsenic (As), is also discussed.

## DNA methylation

### Cadmium

Cadmium is characterized by relatively high mobility and therefore is easily taken up by plants and transported within plant organs. Exposure to this metal causes disturbances in water and mineral homeostasis, decreased chlorophyll levels and diminished photosynthesis efficiency. Cadmium induces oxidative stress and thus leads to an increase in lipid peroxidation and oxidation of proteins and nucleic acids. The adverse effects on genetic material are reflected by elevated levels of micronuclei and chromosome aberrations. Plants exposed to Cd are also characterized by ultrastructural changes, reduced seeds germination and reduced growth (reviewed in Zulfiqar et al., 2021).

The level and pattern of DNA methylation in Cd-treated plants, depends on the plant species, applied metal concentration and treatment duration. The results of MSAP-PCR showed that in Arabidopsis (*Arabidopsis thaliana* L.) treatment with Cd leads to enhanced methylation of external cytosine, while no significant differences were observed in the case of internal cytosines (Wang et al., 2016). In thorn apple (*Datura stramonium* L.) plants methylation sensitive amplification polymorphism analysis (MSAP) revealed that both soil and foliar application of Cd led to an increase in DNA methylation (Shirkhani et al., 2022). In the endangered fern species *Isoetes sinensis*, Cd and Pb induced hemi-methylation, as referred to cytosine methylation on a single DNA strand. On the other hand, a decrease in full-methylation level, referred as methylation of internal and external cytosines on both DNA strands, was observed in metal treated plants (Ding et al., 2018). In soybean (*Glycine max* L.), Cd did not affect the overall DNA methylation level, measured with ELISA assay, neither directly after the treatment nor after seven days of the recovery periods (Holubek et al., 2020). In kenaf (*Hibiscus cannabinus* L.), hybrid F_1_ exhibited higher Cd tolerance and lowest DNA methylation level, when compared with parental cultivars Fuhong 992 (CP085) and P3A (CP089). Among the differentially methylated gene, hypomethylation in the response to Cd was noted in genes encoding leucine-rich repeat receptor-like kinases (*LRR-RLK*), protein belonging to NRT1/PTR family (*NPF2.7*) and DEAD-box ATP-dependent RNA helicase 51 (*DHX51*), while hypermethylation in genes encoding trehalose-phosphate phosphatase (*TTP-D*), NADP-dependent malic enzyme (*NADP-ME*) and NAC-domain containing protein (*NAC71*). The mentioned genes showed also differential expression in response to Cd, although the pattern was not straightforwardly related to the methylation pattern. *LRR-RLK* and *NAC71* were down-regulated, while *NPF2.7, DHX51, TTP-D* and *NADP-ME* were up-regulated (Luo et al., 2023). Another study compared the response of metal hyperaccumulator *Noccaea caerulescens* L. and non-hyperaccumulator, *Arabidopsis thaliana* (Galati et al., 2023). Although *N. caerulescens* accumulated over threefold more Cd than *A. thaliana*, the latter one showed significantly higher degree of DNA damage and higher level of the DNA oxidation marker, 7,8-dihydro8-oxo-2-deoxyguanosine (8-oxo-dG), in reaction to Cd. The results of methy-sens comet assay revealed that Cd induced DNA methylation in *N.caerulescens*, which was accompanied by higher expression of *MET1*. In turn, *A. thaliana* plants were characterized by decrease in DNA methylation, lower *MET1* expression and induction of *DRM2*. It is possible that augmented methylation of DNA observed in the hyperaccumulator contributes to DNA protection and higher metal tolerance. A whole genome bisulfite sequencing (WGBS) of rice (*Oryza sativa* L.) revealed nearly 2,400 differentially methylated regions (DMRs), wherein hypermethylation of the up-stream regions of the genes was the most pronounced response (Feng et al., 2016). The functional analysis of methylated genes showed that most of them belonged to the groups “cellular processes”, “metabolic processes” and “stimuli response”.

The possible changes in DNA methylation pattern might be also reflected by modulated expression or activity of enzymes involved in the methylation or demethylation process. In seagrass, *Zostera marina*, exposure to Cd resulted in increased expression *CMT3* and *DRM*2 methylases and diminished expression of *ROS1* demethylase (Greco et al., 2019), which might results increase in DNA 5mC levels. Transcriptomic analysis of *Brachypodium distachyon* revealed that Cd induced *Bradi4g26905* gene, an ortholog of Arabidopsis *Aly1* and *Aly2*. The encoded ALY proteins are involved in mRNA transport and in DNA methylation (Askoy et al., 2022). Cadmium dependent changes in the expression of DNA methylation regulative genes was also observed in two distinct populations of metal hyperaccumulator *A. helleri*, namely population I16 derived from Italy and population PL22 growing in Poland (Galati et al., 2021). Interestingly, plants belonging to populations I16 and PL22 showed contrasting effects. In the case of PL22, exposure to Cd resulted in increased expression of *MET1*, *WIM1*, *DRM2* genes engaged in DNA methylation as well as genes associated with histone modifications encoding histone-lysine *N*-methyltransferase (KRYPTONITE, KYP), histone H3mK9 demethylase (INCREASE IN BONSAI METHYLATION 1, IBM1) and histone deacetylase 8 (HDA8). The observed changes were accompanied by chromatin condensation. In contrast, population I16 exhibited decreased expression of all of the fore mentioned genes. In Arabidopsis, WGBS analysis revealed that Cd induced DNA methylation, mainly in CHG and CHH contexts and significantly repressed expression of demethylases *ROS1*, *DML2* and *DML3* (Fan et al., 2020). Mutants deficient in *ROS1*, *DML2* and *DML3* expression were characterized by higher metal tolerance and lower Cd uptake. In *Amaranthus cruentus* the expression of eight methylases (*AcDRM2a*, *AcDRM2b*, *AcCMT1*, *AcCMT2a*, *AcCMT2b*, *AcCMT3, AcMET1a* and *AcMET1b*) and two demethylases (*AcDML2a* and *AcDML2b*) was assessed under optimal conditions and in response to metal treatment (Lancíková et al., 2023). The most pronounced response was observed in the roots, which constitute the first site of metal action. Exposure to Cd, Pb and Zn induced expression of all of the analyzed genes. A strong induction of *AcCMT1*, *AcCMT2a*, *AcDRM2b*, *AcMET1* and *AcDML2a* and *AcDML2b* was also noted in response to combined Cd/Zn and/or Cd/Pb treatment. In the case of leaves, the most significant reaction included stimulation of *AcDRM2a* expression under Pb stress. The fore mentioned studies evidence that exposure to Cd is associated with changes in the expression of genes encoding enzymes involved in DNA methylation/demethylation machinery. However, it is not clear whether such changes constitute a regulative role or are rather a side effect of Cd action. In the latter case they might be actually a symptom of metal toxicity leading to massive changes in DNA methylation, which in turn leads to changes in the expression of numerous genes affecting general plant functioning.

The alerted Cd uptake might be dependent on changes in the methylation status and expression levels of transporter genes. In maize (*Zea mays* L.), Cd caused significant increased expression of *Met1*, *Met2a*, *Met2b*, *Met3a*, *Met3b*, *Met3c* and *Met4* methyltransferases accompanied by increased methylation levels of *IRT1*, *ZIP1*, *ZIP2* and *ZIP6* transporters (Shafiq et al., 2020). In wheat (*Triticum aestivum* L.), the response of metal tolerant and sensitive varieties were compared and the metal tolerant variety showed lower methylation levels in the promoter regions of several transporter genes under Cd, Pb and Zn treatment (Shafiq et al., 2019). These changes were accompanied by higher expression of *TaABCC2*, *TaABCC3*, *TaABCC4* and *TaHMA2* transporter genes. In this case, attenuated methylation of transporter genes might have facilitated metal efflux and sequestration in vacuoles leading to enhanced tolerance.

### Lead

According to the Agency for Toxic Substances and Disease Register (ASTDR, https://www.atsdr.cdc.gov/spl/index.html) lead is the next toxic heavy metal/metalloid after arsenic and its role in biological systems has not been fully ascertained. Reported effects of Pb toxicity include perturbation of water balance, reduced seed germination, reduced plant growth, membrane damage, stomata closure and oxidative damage (reviewed in Zulfiqar et al., 2019). Pb toxicity is also reflected by inhibition of several enzymes involved in photosynthesis, nitrogen and nucleic acids metabolism e.g. δ-aminolaevulinate acid dehydratase (ALAD, ribulose-1,5 bis phosphate (Rubisco), phosphoenolpyruvate carboxylase (PEPC), glutamine synthase, nitrate reductase, deoxyribonucleases and ribonucleases (reviewed in Kumar and Prasad, 2018; Zulfiqar et al., 2019).

There are relatively fewer reports on the epigenetics responses to Pb stress in plants. In maize, the impact of Cd, Zn and Pb on the DNA methylation of promoters of selected transporter genes was investigated (Shafiq et al., 2020). The results revealed that Pb exposure resulted in increased methylation levels of *ZIP2* and *ZIP8* promoters. In parallel, decreased expression of histone deacetylases genes (*ZmHD1b*, *ZmHDA102*, *ZmHDA110*, *ZmHD2a*, *ZMHD2b*, *ZmHD2c*, *ZmHDA106*) was observed. The authors suggest that changes in chromatin landscape driven by histone acetylation and DNA methylation of certain promoters modulates expression of specific transporters. This in turn affects mineral uptake and homeostasis in plants. Indeed, the same study showed that Pb treatment led to increased Ca levels in the shoots and Mg levels in the roots and shoots of maize plants. In contrast, decrease in Ca level in the roots and Mg level in the leaves was noted.

The impact of Pb on the methylation and expression of transporter genes was also reported in radish (*Raphanus sativus* L.) plants (Tang et al., 2021). The WGBS revealed 11930 differentially methylated regions (DMRs) in Pb treatment and the control, wherein hypermethylation was the most frequent response. The methylation was mostly observed in the CG context (approx. 49%) followed by CHH (approx. 29%) and CHG (approx. 26%). The methylation had varying effects on genes expression – the downregulation was noted in 76 hypermethylated regions and 38 hypomethylated regions. In turn, 72 hyper- and 42 hypo- DMRs were upregulated. Treatment with Pb resulted in significant changes in methylation and expression of several genes encoding transporters and transcription factors. Increase in the methylation accompanied by hampered expression was noted in the case of *RsABCF5*, *RsABCG14* and *RsZIP11* transporter genes and *RsWRKY46* transcription factor. In turn, decrease in the 5mC levels and enhanced expression was reported in the case of genes encoding MATE efflux protein, heavy metal transport protein (HMT) and ZFP transcription factor.

### Aluminum

Aluminum (Al) is the most abundant metal in the earth’s crust and is considered the major phytotoxic agent in acidic soils as it is highly bioavailable at low pH. The main site of Al action is the plant root. Exposure to this metal leads to cell wall thickening, arrested cell division and consequently retardation of root growth. This in turn results in decreased uptake of nutrients and water. Al induced nutrient deficiency is further augmented by the inhibitory effect of Al on the transporters of some essential ions, for example, calcium (Ca^2+^) and potassium (K^+^). The other symptoms of Al phytotoxicity include leaves yellowing and curling, attenuated photosynthesis and elevated ROS levels (Shetty et al., 2021; Liu et al., 2022).

Plant epigenetic responses to Al was recently reviewed by Gallo-Franco et al. (2020). Changes in DNA methylation levels in response to Al were reported in several plant species including Arabidopsis, wheat, maize, tobacco (*Nicotiana tabacum* L.) and sorghum (*Sorghum bicolor* L. *Moench*). In general, Al exerts DNA demethylation, although opposite effects were also observed (Gallo-Franco et al., 2020). For instance, in triticale lines exhibiting lower Al tolerance a small decrease in DNA methylation was observed (Niedziela, 2018). In contrast, Al tolerant lines showed slight increases in DNA methylation. However, these results were observed using reverse-phase high-performance liquid chromatography (RP-HLPC) and not by methylation-sensitive amplified fragment length polymorphism (metAFLP) or MSAP methods.

In rice, a genome-wide methylation comparison between cultivated (*Oryza sativa*) and the more Al tolerant wild species (*O. glumaepatula*) was undertaken (Gallo-Franco et al., 2022). In the case of *O. sativa*, 4633 differentially methylated regions (DMRs) were observed - 38% showed increased methylation and 62% were hypomethylated. In contrast, in *O. glumaepatula* 72% of the DMRs were hypermethylated and 38% were hypomethylated. In addition, 21 DMR-associated genes (DAGs) in *O. sativa* and 37 in *O. glumaepatula* were associated with Al-responsive genes. Interestingly, about 70 of the DMRs were in TEs that were in close proximity to the Al-responsive genes.

It is worth pointing out that barley plants (*Hordeum vulgare* L.) cope with Al stress by DNA demethylation of retrotransposon insertion in the upstream sequence of the *HvAACT1* (Al-activated citrate transporter1), which enhance *HvAACT1* expression in roots and consequently promote external detoxification of Al (Kashino-Fujii et al., 2018). In general, these studies point to the involvement of DNA methylation machinery in Al-tolerance however, further research is required to understand the specific mechanistic components.

### Mercury

Exposure to metals may lead to long-lasting heritable changes in stress tolerance, which are dependent at least partially in epigenetic modifications. Thus, plants can acquire so called stress memory, which affects not solely their stress reactions but also the reactions of the progeny. For example in Arabidopsis, treatment with Cu and Ni resulted in higher tolerance of the progeny to these metals as well as to methyl methane sulfonate and salt stress (Rahavi et al., 2011). Heritable methylation pattern changes were observed in rice (Ou et al., 2012). In this study, plants exposed to Cu, Cd, Cr and Hg showed hypomethylation of DNA in the CGH context in sequences associated with TE and protein-coding genes. The observed modulation of DNA methylation was accompanied by modified expression of genes involved in chromatin regulation – mostly attenuated expression of genes involved in methylation (*MET1-2*, *CMT3-2*) and induced expression of genes associated with demethylation processes (*DME*). In the case of Hg stress, the possible inheritance of the observed response was assessed. In the first generation after Hg stress, three methylation patterns were observed in the progeny – the same pattern as in parental plants, further hypomethylation or reversion of the hypomethylation. It is worth highlighting that the enhanced hypomethylation was the most frequent pattern. The DNA methylation pattern was inherited, at least partially, by the S1 and S2 generations. In addition to alerted methylation, the second generations of plants showed higher tolerance to Hg reflected by attenuated growth inhibition and chlorophyll loss.

In another study on rice, inheritable changes in the expression of several genes were reported (Cong et al., 2019). Plants treated with Cu, Cd, Cr, Hg showed induced expression of *Tos17*, *Osr42*, *HSP70*, *OsHMA1*, *osHMA2*, *OsHMA5-OsHMA9* and homeobox and elongation factor encoding genes. The expression pattern was at least partially inherited by the progeny in S_1_ and S_2_ generations derived from the Hg-treated plants. To elucidate if the changed abundance of transcripts were associated with DNA methylation, the methylation pattern of Tos17 TE was assessed. The results showed that Hg induced modification of the methylation pattern in relation to the control, which was inherited by the progenies. However, the data indicate that there was no direct interrelation between the pattern of Tos17 expression and methylation. The TE remained inactive in the plants exposed to Hg as well as in the progenies.

The Hg-dependent changes in rice methylome were also assessed using WGBS (Cong et al., 2021). In the study a Hg-sensitive and Hg-resistant lines were used, wherein the Hg-resistant line was selected from the plants with mutation in OsMET1 gene. The sequencing results showed that hypermethylation was the predominant response to Hg. Moreover, changes in CHH context were the most frequent, while CG sites showed the highest stability. In addition, the changes were more dynamic in the Hg-resistant line. The genes differentially methylated under Hg treatment included e.g. genes associated with signaling pathways, stress response and translation process. In general, the cited studies show that Hg affects the status of plants DNA methylation. However, its patterns depends on the applied condition as even in the same plants species, e.g. in rice, some studies show Hg-dependent hypo- while other studies hypermethylation. In addition, the presented data show that methylation pattern can be inherited by next generations of plants and might be associated with their augmented tolerance to metals.

### Arsenic

Arsenic has modest abundance in the Earth’s crust and can occur in various oxidative forms, wherein As^V^ and As^III^ are the most bioavailable and phytotoxic species for plants. The As^III^ is in general more toxic than As^V^, as As^III^ can bind to sulphydryl groups of proteins affecting their structure and functions. As^V^ also negatively affects plants metabolism through competition and replacement of phosphate in cellular pathways. Importantly, As can accumulate in the edible parts of plants, and can severely affect human health. Contamination of water and crops with As is a serious problem in some parts of the world with an estimated 200 000 million people exposed. Prolonged exposure to high levels of this metalloid are associated with several types of cancers including skin, liver, lung, urinary tract, prostate and blood cancer (Genchi et al., 2022; Martínez-Castillo et al., 2022).

The effects of As exposure also include epigenetic changes. For example, in the hyperaccumulator Cretan brake fern (*Pteris cretica* L.) As causes decreased DNA methylation (Zemanová et al., 2020). This response was observed only in old fronds. In contrast, in young fronds the methylation status was unchanged and was independent of the applied As concentration. There are premises, that similarly to metals, also As uptake is regulated by DNA methylation. The overexpression of *VIM1* (Variant In Methylation/Orthrus), which is a methylcytosine binding protein, led to higher As tolerance and lower levels of this metalloid in *A. thaliana* (Bahmani et al., 2023). In contrast, *VIM1* deficient mutants showed higher As accumulation and more severe toxicity symptoms than the wild type plants. A more detailed analysis showed that VIM1 mediates DNA methylation of the promoters of transporter genes, leading to their decreased expression and associated reduced As uptake.

The impact of metals and metalloids on DNA methylation levels and patterns is summarized in **Table 1**.

**Table 1:** Examples of essential and non-essential metal treatments on DNA methylation levels, CMT – chromomethylase, DMGs – differentially methylated genes, DML - Demeter-like protein, DRM - domains rearranged methylase, IBM - histone H3mK9 demethylase, HDA - histone deacetylase, KYP - histone-lysine *N*-methyltransferase, MET – methyltransferase, ROS1 - repressor of silencing, VIM1 - Variant In Methylation/Orthrus.

Metal or metalloid	Concentration; treatment duration	Plant species	Effect	References
Cd, Pb	Cd: 100, 250 and 500 mg/l, Pb: 500, 1000 and 2000 mg/l; 10 days	*Isoetes sinensis* L.	↑ CCGG hemi-methylation ↓ CCGG full-methylation	Ding et al., 2018
Cd	75, 150 and 225 ppm two months	*Datura stramonium* L.	↑methylation ↓demethylation	Shirkhani et al., 2022
Cd	0.89 and 8.9 µM; 6 days	*Zostera marina*	↑expression of *CMT3* ↑expression of *DRM2* ↓expression of *ROS1*	Greco et al., 2019
Cd	5 μM for 5 days (*A.thaliana*); 50 μM for 14 days (*A.halleri*)	*Arabidopsis thaliana* L., *A. helleri* L.	↓expression of MET1 and DRM2 encoding genes in *A. thaliana* ↑expression of *MET1* and *DRM2* in *A. halleri* population PL22 accompanied by increased CpG methylation ↓ expression of *MET1* and *DRM2* in *A.halleri* population I16 accompanied by decrease in CpG methylation	Galati et al., 2021
Cd	5 μM in *A. thaliana*, 50 μM in *N. caerulescens* 7 days	*A.thaliana* *Noccaea caerulescens* L.	↑ DNA methylation accompanied by higher DNA damage in *A. Arabidopsis* ↓DNA methylation accompanied by lower DNA damage in *N. caerulescens*	Galati et al., 2023
Cd	80 μM for 4 days	*Oryza sativa* L.	↑CHH methylation ↓CHG and CG methylation nearly 2400 DMRs	Feng et al., 2016
Cd	40 μM for 7 days	*A. thaliana*	↑DNA methylation, mainly in CHH and CHG context modulated expression of genes engaged in regulation of methylation process ↓expression of ROS1, DML2 and DML3 demethylases	Fan et al., 2020
Cd, Pb	100 μM for 2 days	*Triticum aestivum* L.	modulated expression of DNA methylation associated genes in metal tolerant variety - ↓methylation of promoter regions in transporter genes	Shafiq et al., 2019
Cd, Pb	100 μM for 2 days	*Zea mays* L.	alerted methylation level of the promoter regions of IRT and ZIP transporters	Shafiq et al., 2020
Cd, Pb, Cd+Pb	Cd 15 mg/l, Pb 200 mg/l, Cd+Pb (15 + 200 mg/l)	*Amaranthus cruentus L.*	↑expression of genes from *CMT1-3*, *DRM2*, *MET1* and *DML2* families	Lancíková et al., 2023
Pb	200 mg/l	*Raphanus dativus* L.	changes in methylation level with predominant hypermethylation, changes in methylation level and expression pattern of several transporter and transcription factor encoding genes	Tang et al., 2021
Al	15, 20 and 30 ppm; pH= 4.5 24 h	triticale	↑global methylation in Al-tolerant lines ↓global methylation in Al-sensitive lines	Niedziela, 2018
Hg	50 and 1000 µM; 7 days	*O. sativa*	transgenerational changes in methylation pattern	Ou et al., 2012
Hg	50 and 1000 µM; 7 days	*O. sativa*	changes in the methylation level/pattern of *Tos17* transposable element observed in S_0_, S_1_ and S_2_ generations	Cong et al., 2019
Hg	100 µM; 30 days	*O. sativa*	↑methylation of DNA, mostly in CHH context DMGs included genes engaged in signalling, stress response and translation	Cong et al., 2021
As	100 and 250 mg/kg of soil; 122 days	*Pteris cretica* L.	↓in global methylation in old fronds no changes in methylation status in young fronds	Zemanová et al., 2020
As	35 μM; 21 days	*A. thaliana*	overexpression of *VIM1* leads to higher methylation in the promoters of transporters genes and decreased As uptake	Bahmani et al., 2023

## Histone modifications

Exposure to non-essential metals modulates expression of histones onvarious levels including transcript and protein abundance. For example in barley, metal sensitive line was characterized by Cd-dependent decreases in the level of transcripts encoding H2A, H2B, H3 and H4 histones, whereas no changes were observed in the metal tolerant line (Cao et al., 2014). In soybean cell suspension, Cd induced accumulation of H2A histone protein (Sobkowiak and Deckert, 2006). In addition, metals modulate the expression of genes involved in histone modifications. For example, elevated expression of several genes involved in histone acetylation and deacetylation was reported in cotton (*Gossypium* L.) in response to Cd, and in maize in response to Cd and Pb (Imran et al., 2019; Imran et al., 2020; Shafiq et al., 2020). Interestingly, the expression pattern can vary within different lines of the same plant species. Treatment with Cd caused increases abundance of transcripts encoding KYP, IBM1 and HDA8 in *A. halleri* PL22 population but decreased abundance in the I16 population (Galati et al., 2021). Changes in histone modifications were also evidenced by immunofluorescence techniques in root meristem cells of broad bean (*Vicia faba*) (Żabka et al., 2021). Decreased demethylation of histone 3 at lysine 79 (H3K79Me2), which is associated with replication initiation events, was observed in Cd-treated plants in relation to the control in all three analyzed cell cycle phases (G1, S and G2). In addition, exposure to Cd resulted in attenuated phosphorylation of histone 3 at threonine 45 (H3T45Ph), a modification engaged in the processes of DNA replication. In contrast, increased levels of histone 3 acetylation on lysine 56 (H3K56Ac) was observed in Cd-treated plants at S cell cycle phase.

The possible role of histone modifications during metal stress was also reported in mutant plants. The Jumonji-C-domain-containing protein (JMJ) belongs to histone demethylases. Increased expression of the *SIJMJ524* gene was noted in tomato plants under Hg, Pb and Cu treatment (Li et al., 2022). Its overexpression in Arabidopsis modulated tolerance towards Cd. The young seedlings showed decreased, while the older plants enhanced tolerance. Under Cd treatment, the *SIJMJ524* over-expressing plants showed higher expression of genes engaged in metal uptake (*ZIP1*, *IRT1*, *COPT2*, *NRAMP1*) and transport (*ABCC1*, *PDR1*, *GSH1*, *GSH2*). However, some transport and detoxification related genes showed attenuated expression e.g. genes encoding phytochelatin synthase (*PCS1*, *PCS2*) and glutathione reductase (*GR1*). Phenolic compounds constitute an abundant group of secondary metabolites engaged in plant stress defense e.g. through participation in metal binding, lignin synthesis and wall thickening or antioxidant functions (Anjitha et al., 2021). Over-expression of SIJMJ524 resulted in modulation of phenolic biosynthesis. The mutant plants accumulated higher levels of flavonoid, which was accompanied by increased expression of *PAL* (*phenylalanine ammonia lyase*) and *FLS* (*flavonol synthase*) genes. On the other hand the *DFR* (*dihydroflavonol 4-reductase*) and *CHI* (*chalcone isomerase)* expression was decreased (Li et al., 2022).

## Epitranscriptomic changes

Even though the first RNA modifications in plants were described 70 years ago, research progress was slow until newly developed techniques were applied over the last decade. Recent studies showed that modified ribonucleotides can modify the half-life of transcripts, translation efficiency and translocation and within the cell and plant organs (reviewed in Chmielowska-Bąk et al., 2019). However, relatively little is known about the role of RNA modifications in plant stress responses, particular to metals and metalloids.

Methylation of adenosine at N6 position (m6A) is recognized as the most frequent and widespread modification of ribonucleotides in plants. Its profound significance during plant development is highlighted by the fact that Arabidopsis mutants in the adenosine methylation machinery are embryo-lethal (Bodi et al., 2012). In mature Arabidopsis plants, attenuated methylation of adenosine results in perturbed development of vascular bundles, trichomes, roots, rosette leaves, roots and flowers (Bodi et al., 2012). Recently, a link between m6A and senescence was identified (Rudy et al., 2023). Alerted m6A patterns were recently reported in the plant’s response to stress (Tang et al., 2023). In the case of metal stress, m6A was studied in soybean after Cd and Pb treatment and in barley exposed to Cd (Su et al., 2022; Han et al., 2023; Zhang et al., 2023). In barley, a 7-day long exposure to Cd generally resulted in adenosine hypermethylation. Among the 435 differentially expressed genes (DEGs), the majority (319 genes) showed increased methylation and increased RNA abundance. The up-regulated, methylated transcripts encoded proteins engaged in signalling (MAPK signalling, hormone signal transduction), phenylpropanoid pathway, sucrose and starch metabolism, glycolysis and gluconeogenesis and transport (ABC transporters). GO analysis revealed that the DEGs were involved in oxidation-reduction processes, transferase and oxidoreductase activity and transmembrane transport (Su et al., 2022). Similarly in soybean, treatment with Cd resulted in increased levels of m6A in transcripts. The methylated transcripts were associated with stress and signalling pathways – plant hormone signalling, MAPK pathway and pathogen interaction. In addition, the m6A bearing transcripts were enriched in the metabolism pathways for sucrose and starch, fatty acids, amino acids and aminoacyl-tRNA. The hypermethylated and down-regulated genes included genes involved in polyamine catabolism. The authors suggest that reduced expression of these transcripts leads to the accumulation of polyamines and might constitute one of the stress protective mechanisms (Han et al., 2023). Interestingly, in animal studies several links between transcript methylation and histone modifications were found (reviewed in Xu et al., 2022). For example, it was shown that H3K36me3 is recognized by transcript methylation machinery leading to m6A deposition in newly transcribed RNAs. In addition, several cases of relation between the expression of proteins engaged in histone modifications and transcript methylation were discovered. It was also found that interaction between m^5^C and its methylation machinery might impact chromatin conformation (Cheng et al., 2018).

One of the universal consequences to stress, including metals, is perturbed ROS homeostasis, which can lead to oxidative damage (Riyazuddin et al., 2021; Mittler et al., 2022). On the other hand, ROS can also act as a signalling molecule engaged in stress signal transduction and regulation of genes expression (Peláez-Vico et al., 2022). Oxidation can also occur on ribonucleic acids resulting in the formation of 8-hydroxyguanosine (8-OHG). In soybean seedlings, short term treatment with Cd and Pb induced 8-OHG accumulation in total RNA and/or poly(A) RNA. The observed response was rapid, observed only after few hours of metal exposure, and preceded the symptoms of oxidative stress (Chmielowska-Bąk et al., 2018; Chmielowska-Bąk et al., 2022). However, the exact role of this early 8-OHG formation in plants is still to be revealed.

Some studies indicate that transcript modifications may play a significant role in the plant’s responses to metals. This hypothesis is supported by the fact that other RNA modifications, namely 5-methylcytosine (m^5^C) and 8-nitorguanosine (8-NO_2_-G), were implied in plant stress responses. Knock-outs of RNA m^5^C methyltransferases resulted in decreased m^5^C levels and higher sensitivity to oxidative stress in Arabidopsis and to reduced heat tolerance in rice (David et al., 2017; Tang et al., 2020). In the case of rice, the mutants were characterized by lower expression of photosynthesis-related genes. The authors suggest that altered m^5^C levels on transcripts leads to the inability of the photosynthesis apparatus to adapt to stress conditions. In addition, disfunction of the photosynthesis process leads to enhanced accumulation of ROS and to formation of oxidative dependent lesions (Tang et al., 2020). In the case of 8-NO_2_-G, increased levels of this modification was observed in total RNA and mRNA of potato plants in response to the inoculation with the pathogen *Phytophthora infestans*. Interestingly, the response was significantly higher in the *Phytophthora* resistant potato cultivar when compared to the susceptible cultivar, suggesting that 8-NO_2_-G accumulation in the transcripts is associate with defense mechanisms (Izbiańska et al., 2018). Interestingly, 8-NO2-G formation was also observed in the phytopathogen structures (*P. infestans*), and exposure to sub-lethal Cd concentration induced this type of RNA modification (Gajewska et al., 2020).

The transit nature of most RNA modifications, as well as the rapid turn-over of the modified transcripts, indicates that ribonucleotide modifications may act as fast switches adjusting the plants metabolism to changes in both developmental and environmental stimuli. However, this hypothesis requires more experimental support.

## The relation of epigenetic and epitranscriptomic changes to metal tolerance

The fore mentioned studies evidence that non-essential metals and metalloids, namely Cd, Pb, Hg, Al and As, affect the level and pattern of DNA methylation and histone and transcripts’ modifications, thus inducing epigenetic and epitranscriptomic changes. There are convincing premises indicating that these changes can be related to the enhancement of metal tolerance. For example, the metal hyperaccumulator *Noccaea caerulescens* showed higher DNA methylation level and lower DNA damage when compared with more sensitive but closely related plant species, *Arabidopsis thaliana* (Lancíková et al., 2023). Differential methylation patterns and higher metal tolerance was also evidenced in kenaf F_1_ hybrid when compared with the parental cultivars (Luo et al., 2023). In addition, research on Arabidopsis revealed that tolerance towards metals can be enhanced by the inhibition of the DNA demethylation process (Fan et al., 2020).

There might be several protective mechanisms dependent on the epigenetic and epitranscriptomic changes. Firstly, methylation might affect DNA’s susceptibility to the damage. Arabidopsis cell suspension cultures deficient in methylation showed higher rate of single base substitutions (SBS) when compared to the control. The effect was further augmented under salt stress. The results indicate that DNA methylation might exert protection against mutations (Zhu et al., 2021). In addition, a connection between histone modifications and the DNA damage recognition and repair has been described (reviewed in Gong and Miller, 2019).

Secondly, it was shown that genomic regions differentially methylated under metal treatment include transporter genes (e.g. Bahmani et al., 2023, Cong et al., 2021, Feng et al., 2016; Tang et al., 2021). In Arabidopsis over-expression of VIM1 proteins leads to enhanced methylation of the promoter region of the *NIP3;1* transporter gene leading to its decreased expression, hampered As uptake and consequent enhanced growth parameters under As treatment (Bahmani et al., 2023). Similar methylation-dependent mechanism might also function in plants response to other metals. In addition, the expression of transporter genes might be modulated by the methylation of transcripts. In barley, treatment with Cd resulted in hypermethylation and simultaneous induced expression of genes encoding ABC and zinc transporters (Su et al., 2022).

Lastly, DNA as well as RNA methylation leads to the regulation of the expression of stress signalling and defense associated genes. The WGBS analysis revealed thousands of differentially methylated regions in plants in response to Cd, Pb, Al and Hg treatment (Cong et al., 2021, Feng et al., 2016; Gallo-Franco et al., 2022; Tang et al., 2023). In rice, the majority (nearly 70%) of Cd responsive genes were also differentially methylated. In addition, some genes involved in stress response and detoxification processes, namely genes encoding lipoxygenases (*LOX*) or glutathione S-transferases (*GST*, *GSTU 35*), were characterized by Cd-dependent increase in methylation and transcript levels (Feng et al., 2016). Another study on rice showed that under Hg treatment, genes associated with oxidative response were hypomethylated in Hg-resistant and hypermethylated in susceptible lines (Cong et al., 2021). In kenaf F1 hybrid, characterized by higher Cd tolerance, exposure to the metal resulted in decreased methylation and enhanced expression of *NPF2.7* gene, encoding protein belonging to NRT1/PTR family involved in nitrate, amino acids and plant hormones transport. Silencing of the *NPF2.7* gene resulted in greater growth inhibition and augmented oxidative stress in reaction to Cd, highlighting its important in plant metal tolerance (Luo et al., 2023). Changes in the methylation pattern of transcripts might also affect the expression of genes (Su et al., 2022; Han et al., 2023). In barley, m6A enrichment is mostly associated with higher transcripts abundance (Su et al., 2022). Exposure to Cd resulted in hypermethylation of transcript encoding glutathione S-transferase and stress associated transcription factors belonging to WRKY and MYB families, which was accompanied by their elevated levels. In the case of MAPK and peroxidases encoding genes, in most cases similar pattern was observed, although in individual genes also hypomethylation and decreased transcripts levels were reported. On the other hand in soybean, 50% of differentially methylated and expressed transcripts showed hypermethylation and down-regulation (Han et al., 2023). The hypermethylated and down-regulated genes included genes encoding thioredoxin peroxidases, peroxidases, polyamine oxidase and gens associated with plant hormone and calcium signalling.

Importantly, the metal dependent changes in the pattern of DNA methylation and genes expression can be inherited by the next generations affecting their future stress response (Ou et al., 2012; Cong et al., 2019). In rice the DNA methylation pattern observed under Hg treatment was, at least partially, transmitted to the S_1_ and S_2_ generations. In addition, plants were characterized by inheritable changes in genes expression and higher tolerance towards the metal.

However, it is worth highlighting that the tolerance associated changes in DNA methylation are rather limited to specific loci. On the other hand the global, whole-genome changes can be a result of abnormal expression of genes involved in methylation/demethylation. The consequences of such changes would include massive alterations in genes expression and disturbed plant functioning. In addition, it is postulated that global modulation of DNA methylation might alert genomic stability and lead to rearrangements of chromosomes. In humans, global loss of methylation is associated with the development of facial anomalies syndrome (ICF). Alterations in DNA methylation are also observed by other diseases including cancer, cardiovascular disorders, type II diabetes, lupus and schizophrenia (reviewed in Szyf, 2011). Thus, it is possible that in plants metals act as “toxicomethylomic” agents, affecting expression of genes engaged in methylation/demethylation processes. This in turn leads to alterations in global methylation status, disturbed expression of numerous genes and alerted genomic stability.

## Conclusions

A significant number of publications provide evidence that non-essential metals and metalloids affect cell’s DNA methylation status. However, the exact pattern of observed DNA methylation changes is dependent on various factors including plant species, applied metal, its concentration and the treatment duration. In addition, significant differences were observed between the metal sensitive and tolerant lines e.g. in triticale or rice or even between the parental plants and the F_1_ hybrid (**Table 1**). Metal impact on histone modifications is relatively less well studied, however on-essential metals were shown to modulate expression of gene’s associated with histone biosynthesis and modifications on the transcriptional and post-transcriptional levels (Cao et al., 2014, Deckert and Sobkowiak, 2006, Imran et al., 2019; Imran et al., 2020; Shafiq et al., 2020). Conseuquenlty, also the pattern of histone modifications (methylation and acetylation) was modulated by the metals (Żabka et al., 2021). Epitranscriptomic changes are relatively new players in the field of plant stress biology. Recently, the first epitranscriptomic reports revealed that exposure to Cd modulated the pattern of transcript m6A methylation in barley and soybean plants (Su et al., 2022; Han et al., 2023). Treatment with Cd and Pb also lead to early increases in the level of oxidation dependent RNA modification –8-hydroxyguanosine (8-OHG), although the role of 8-OHG in metal response is still to be elucidated (Chmielowska-Bąk et al., 2018; Chmielowska-Bąk et al., 2022).There are premises indicating that epigenetic and epitranscriptomic changes contribute to the enhancement of plant metal tolerance e.g. through protection of DNA from damage, modulation of the expression of genes related to metal transport, cellular signalling and stress defense leading to hampered metal uptake and more efficient inter-cellular distribution as well as to accumulation of protective compounds (e.g. flavonoids).

**Figure 1:**
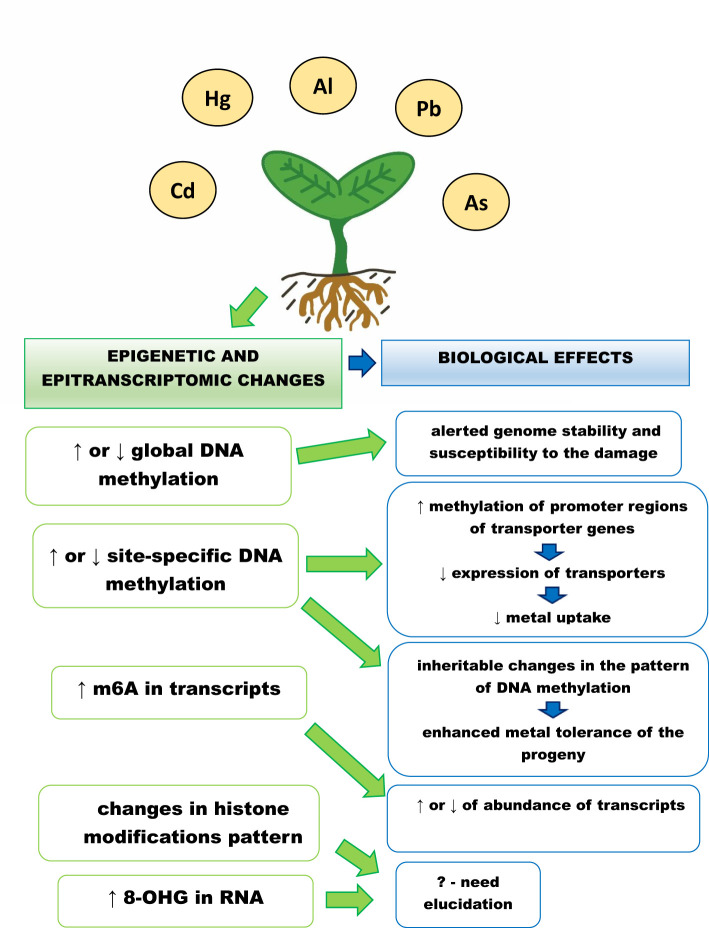
Epigenetic and epitranscriptomic responses of plants to metals, ↑ - increase in the level, ↓ - decrease in the level, m6A – adenosine methylated at N6 position, 8-OHG – 8 – hydroxyguanosine. Exposure to metals leads to changes in global and site specific DNA methylation levels, which are dependent on plant species, applied metal, metal concentration and treatment duration. The increase in methylation of transporters promoter regions leads to decrease in their expression and attenuated metal uptake. In addition, changed DNA methylation pattern can be inherited by the progeny and might be associated with its enhanced metal tolerance. Metal exposure also causes changes in histone modification pattern and increase in the level of RNA modifications – m6A and 8-OHG. The exact role of these changes is still not clear. However, increase in m6A is associated with higher abundance of the methylated transcripts engaged e.g. in signalling, transport and carbohydrates metabolism.

The plant’s epigenetic and epitranscriptomic responses to metals is summarized in **Figure 1**. Although significant progress has been made in the understanding of the role of epigenetic and epitranscriptomic changes in plants in response to metals, including non-essential metals, there are still many open questions.

Firstly, in most of the cases it is not clear if the epigenetic changes are a symptom of metal toxicity or regulatory effect associated with acquisition of stress tolerance. On the one hand, there are premises pointing out to the involvement of epigenetic and epitranscriptomic modifications of specific regions or transcripts in the activation of signalling and stress defense network. In addition, studies evidence that methylation of specific DNA regions affects the expression of transporter genes and may regulate metal uptake and intracellular deposition. On the other hand, metal dependent alterations in the expression of genes associated with methylation/demethylation processes may lead to global changes in the level of DNA methylation and, in consequence, to massive changes in genes expression. This would alert normal plant functioning and would be perceived as toxicity symptom. Moreover, global changes in the DNA methylation level affect the stability of genetic material and might lead to its higher susceptibility to the damage. Likely, beneficial or adverse effects of epigenetic and epitranscriptomic modifications are dependent on several internal and external factors, including plant species, developmental stage, the type of applied metal and stress intensity. The fore mentioned hypothesis need experimental support.

Secondly, the pathways leading to the modulated expression of genes engaged in DNA methylation/demethylation processes are not elucidated, although there are premises pointing to the action of ROS and RNS. A study on pokeweed (*Phytolacca americana*) showed that the application of an NADPH oxidase inhibitor, which is a major component of ROS generation, resulted in decrease expression of *CMT2*, *CMT3*, *MET1* and *ROS1* (Jing et al., 2022). Also RNS might exert regulatory function on both histone modifications and DNA methylation status (Rudolf et al., 2021). For example, nitric oxide-related impact on genes regulating DNA (de)methylation, being in a dialog with histone methylation was recently documented in potato immunity (Drozda et al., 2022). In turn, nitric oxide induced histone hyperacetylation at genes related to stress response by inhibiting histone deacetylase activity was evidenced in Arabidopsis (Mengel et al., 2017).

The DNA methylation can be mediated by microRNAs (miRNAs). Metals induce up- or down-regulation of numerous miRNAs in several plant species including *Arabidopsis thaliana*, *Phaseolus vulgaris*, *Medicago truncatula*, *Brassica napus*, *Oryza sativa* and *Nicotiana tabacum*. Under metal stress miRNAs are indicated as elements involved in the regulation of metal uptake and chelation and in signalling, gene regulatory and detoxification processes (reviewed in Gielen et al., 2012; Ma and Hu, 2023). However, the role of these RNA species in metal-dependent methylation is still unrecognized. This area would need further exploration, especially taking into account the fact, that miRNA can modulate DNA methylation under other stress conditions. In barley, one of the drought induced 24mer targets the *HvCKX2.1*. promoter. Further analysis revealed that drought leads to increased methylation of *HvCKX2.1* pointing out to the possible miRNA dependent methylation process (Surdonja et al., 2017).

Thus, more experimental work is needed to get deeper insights into the mechanisms and roles of epigenetic and epitranscriptomic modifications in plants exposed to metals. The outcomes could lead to the development of more tolerant plants through regulation of epigenetic and/or epitranscriptomic pathways or selection of plants with specific pattern of epigenetic modifications for further breeding.

## Author contributions

JC-B: Conceptualization, Funding acquisition, Visualization, Writing – original draft, Writing – review and editing. IS: Conceptualization, Writing – review and editing. TW: Writing – original draft, Writing – review and editing. MA-J: Conceptualization, Writing – original draft, Writing – review and editing.
